# Case report: Intrafraction dose-guided tracking for gastrointestinal organ-at-risk isotoxicity delivery on an MR-guided radiotherapy system

**DOI:** 10.3389/fonc.2024.1357916

**Published:** 2024-07-11

**Authors:** Sreenija Yarlagadda, Yonatan Weiss, Michael David Chuong, Nema Bassiri, Alonso N. Gutierrez, Rupesh Kotecha, Minesh P. Mehta, Kathryn Elizabeth Mittauer

**Affiliations:** ^1^ Department of Radiation Oncology, Miami Cancer Institute, Baptist Health South Florida, Miami, FL, United States; ^2^ Herbert Wertheim College of Medicine, Florida International University, Miami, FL, United States

**Keywords:** MRgRT, dose-guided tracking, SBRT, ablative dose, GI OAR

## Abstract

In the current era of high-precision radiation therapy, real-time magnetic resonance (MR)-guided tracking of the tumor and organs at risk (OARs) is a novel approach that enables accurate and safe delivery of high-dose radiation. Organ tracking provides a general sense of the need for daily online adaptation but lacks precise information regarding exact dosimetry. To overcome this limitation, we developed the methodology for monitoring intrafraction motion with real-time MR-guided isodose line-based tracking of an OAR in combination with anatomic tumor-based tracking and reported the first case treated with this approach. An isolated para-aortic (PA) nodal recurrence from carcinosarcoma of the endometrium was treated with an ablative dose of 50 Gy in five fractions using MR-guided radiotherapy (MRgRT). This report demonstrates the feasibility, workflow, dosimetric constraints, and treatment paradigm for real-time isodose line-based OAR tracking and gating to enable an isotoxicity delivery approach. This innovative treatment strategy effectively tracked the intrafraction motion of both the target and OAR independently and enhanced the accuracy of structure localization in time and space with a more precise dosimetric evaluation.

## Introduction

Radiation dose escalation has been highly effective in achieving high rates of durable local control for several tumors, which can further cause improved overall survival (1–3). However, the delivery of ablative doses is often compromised by the proximity of certain organs at risk (OARs), and this has been a major limiting factor, especially when intrafraction organ motion adds a layer of dosimetric uncertainty, as is frequently observed in the thorax and abdomen. Given the lack of correlation between surface changes and internal organ position, location, and movement, patient surface anatomy cannot be used as a reliable surrogate for the internal motion of the thorax and upper abdominal organs (4).

Real-time tracking and automatic gating of radiation delivery is an effective solution to spare the OARs and mitigate treatment-related toxicity. This enables the safe delivery of ablative doses to the gastrointestinal (GI)/pelvic structures, which was previously impractical due to limited visualization and intrafraction motion. Magnetic resonance-guided radiation therapy (MRgRT) enables online adaptation to customize the radiation to the anatomy of the day, addressing both intra- and interfraction anatomic variation while providing excellent soft tissue visualization. The ability to continuously track in real time with MR imaging addresses the uncertainty associated with intrafraction motion (5), while eliminating the need for invasive procedures such as internal fiducials (required for x-ray-based real-time tracking) or electromagnetic transponders (6). It also eliminates the need to expand the true target to account for motion using the concept of an internal target volume (ITV) and thereby reduces the required setup margin, therefore exposing less normal tissue to unnecessary radiation doses (7).

In contrast to conventional radiation therapy, where homogeneous target coverage is the primary objective, isotoxic dose escalation increases the dose to the target volume until the pre-selected adjacent OAR dose constraint is reached. An isotoxic approach is generally applied when certain OARs are in proximity to the target volume, and these OARs are constrained to a lower dose level than the target volume. As such, this isotoxicity approach is characterized by heterogeneous target coverage, with a higher dose covering the core of the target [usually the gross tumor volume (GTV)] (8). Furthermore, this approach necessitates ensuring a rapid dose falloff of the ablative doses from the core to the periphery of the target, so as to further minimize OAR doses in close proximity. Because of the anatomically constrained dosimetric gradients between the OARs and target volumes in such cases, it is essential to ensure that the motion of the OAR-target geometry is minimized and/or accounted for in isotoxicity planning and delivery.

Liu et al. implemented a multitarget MLC-based motion tracking system for MRgRT that can simultaneously track two independently moving structures and gate the radiation beam in real time, compensating for motion (9). This approach can be used to track the tumor and adjacent dose-limiting OARs concurrently. Such an approach makes isotoxic dose escalation in real time feasible, thus broadening the therapeutic window. MRgRT provides continuous real-time cine MR imaging of the radiation field and is deployed utilizing a simple target margin, i.e., a boundary, for standard anatomic tracking and gating. The addition of isodose line-based tracking can further enhance the intrafraction motion accuracy beyond the use of simple anatomic margin expansion, as has been used historically in MRgRT.

This is the first report to describe the feasibility and workflow of this innovative approach to dose-guided tracking of GI OAR using MRgRT. Additionally, we report the institutional dose constraints for abdominal and pelvic targets to ablative doses (50 Gy in five fractions) and time analysis for the current workflow.

## Case description

A 63-year-old woman was initially diagnosed with stage IB carcinosarcoma of the endometrium in September 2020. She was treated with robotic total laparoscopic hysterectomy with bilateral salpingo-oophorectomy and bilateral pelvic sentinel lymph node dissection, followed by high-dose-rate intracavitary brachytherapy to the upper vagina (21 Gy in three fractions of 7 Gy prescribed to 0.5 cm depth), and completed six cycles of adjuvant carboplatin/paclitaxel chemotherapy in February 2021. A follow-up positron emission tomography–computed tomography (PET‐CT) scan in June 2022 revealed an oligometastatic disease in the left para-aortic (PA) node (1.1 cm), which has further enlarged to 1.3 cm by October 2022, with an increase in maximum standardized uptake value (SUV_max_) from 5.75 to 10.9.

A multi-disciplinary discussion at our institutional gynecology tumor board recommended treating isolated nodal recurrence with ablative radiation therapy. Clinically, she reported intermittent rectal pain and sporadic rectal bleeding, with the last occurrence 1 month before. She was also diagnosed with co-existing acute inflammatory bowel disease (IBD), which raised the concern of severe acute GI toxicity if treated with conventional abdominal and pelvic radiation fields. Therefore, online adaptive stereotactic body radiation therapy (SBRT) was recommended at a dose of 50 Gy in five fractions on the MRIdian system (ViewRay, Cleveland, OH, USA). The MRIdian system combines a 0.35-T MR scanner with a 6-MV flattening filter-free Linac and a double-stacked, double-focused MLC offset by half a leaf width, enabling a leaf resolution of 4.15 mm at the isocenter (10). The patient was included in an institutional review board (IRB)-approved protocol.

## Treatment description

### Simulation and initial planning

The simulation was performed under mid-inspiration breath hold in the supine position with both arms at her sides to ensure patient comfort. The planning MR scan was acquired on the MRIdian at 50 × 50 × 35.8 cm^3^ field of view (FOV) with a resolution of 1.5 × 1.5 × 3.0 mm^3^. The planning MR scan acquired for simulation and also the scan at each fraction for adaptive re-planning were a balanced steady-state free precession (TrueFISP) sequence with a maximum distortion of <1.0 mm and <2.0 mm within 5 cm and 17.5 cm of the isocenter, respectively (11). Patient devices included a foam pad and wing board since continuous MR imaging is available for motion management.

**Figure 1:**
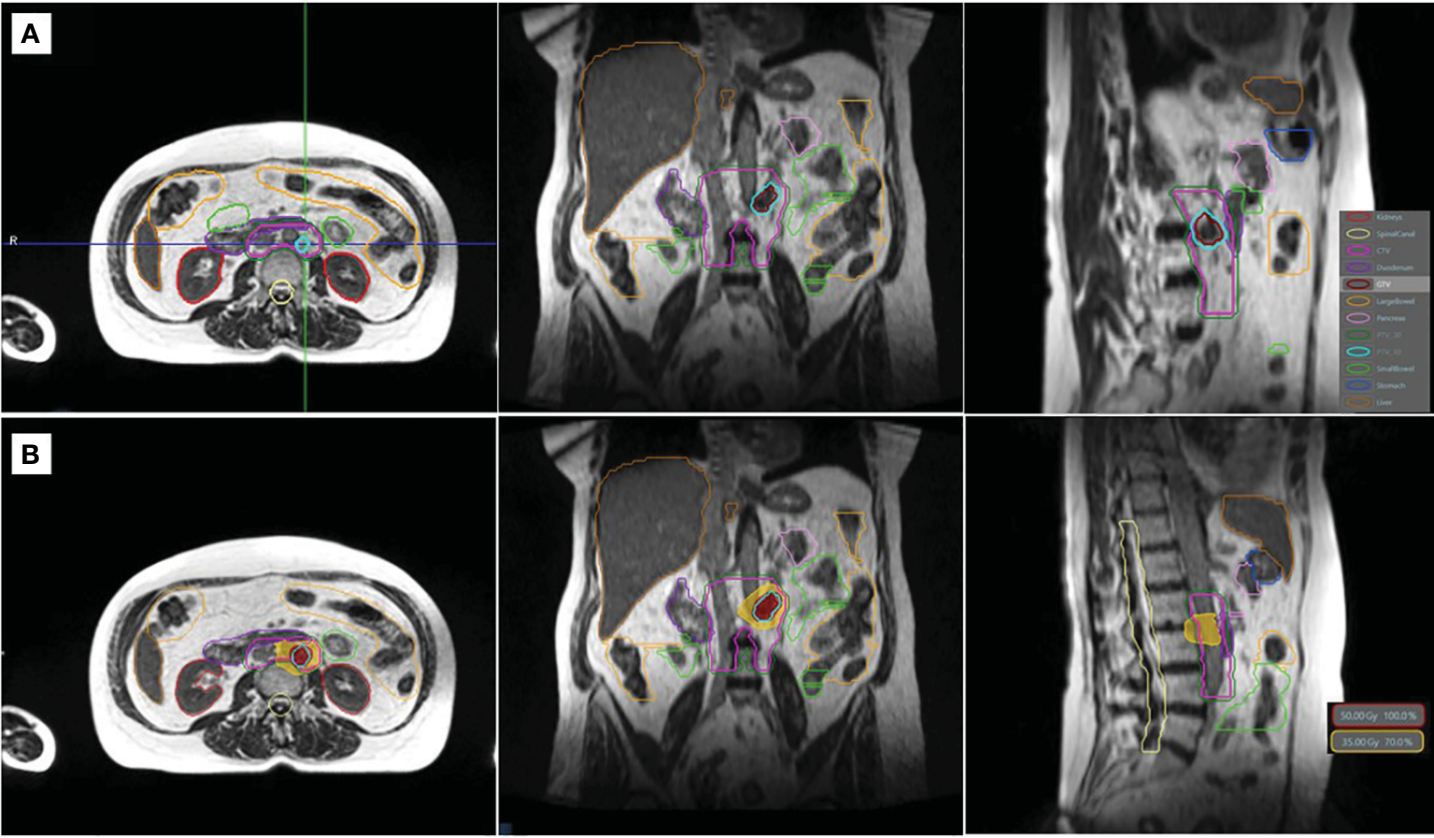
TrueFISP MR scan showing the simulation anatomy **(A)** and the dose distribution of the original plan **(B)**. Relevant organs at risk and targets are shown in outline **(A)**. The isodose lines of the prescription (50 Gy) and gastrointestinal organ-at-risk constraint (35 Gy) are shown in colorwash **(B)**.

Segmentation and treatment planning were performed on the MR simulation scan. The GTV was defined as the tumor visualized on the TrueFISP MR, and the clinical target volume (CTV) was delineated as GTV with a 2-mm isotropic margin, excluding extension into the vertebral body posteriorly, and then uniformly expanded by a 3-mm margin to create the planning target volume (PTV), which was prescribed to 50 Gy (PTV_50_). A simultaneous integrated boost (SIB) technique was used, and a second CTV prescribed to 30 Gy (CTV_30_) was delineated by the PA chain as defined from the renal vessels (superiorly) to the aortic bifurcation (inferiorly), following the RTOG consensus (12). A 3-mm uniform setup margin expansion of the CTV_30_ was used to create the PTV_30_. Relevant OARs segmented included the duodenum, small bowel, large bowel, stomach, kidneys, liver, spinal canal, and cauda equina. **Figure 1** depicts the simulation anatomy and dose distribution on the TrueFISP MR scan.

Our planning technique, which has been previously described in the literature, utilized subdivision of the PTV_50_ to differential dosing based on a nonoverlapping region of the PTV_50_ with a GI planning OAR volume (PRV) (8). To this end, a GI PRV was created as an optimization structure to define the dose falloff between the proximal GI OARs and the target. The GI PRV was defined by a 3-mm isotopic expansion of the union of the stomach, duodenum, small bowel, and large bowel. Any overlapping portion of the GTV and PTV_50_ by the GI PRV was optimized to achieve 25–35 Gy. The non-overlapping portion of the GTV and PTV_50_ with the GI PRV was defined as GTV_opt_ and PTV_50opt_, respectively, and optimized to achieve at least 50 Gy. Further details on the optimization and adaptive robustness of this planning technique have been previously reported (8).

A 15-field step-and-shoot intensity-modulated radiation therapy (IMRT) arrangement with 40 segments was created with a Monte Carlo dose calculation algorithm using a 2-mm isotropic dose grid size. A deformed CT approach was used to map the electron density. Manual edits were applied as needed to correct for GI luminal gas as air and/or tissue. Note that the CT scan was performed in mid-inspiration breath hold and acquired on the same day as the simulation MR scan.

A recent hardware and software upgrade to the MRIdian system was released for clinical use (A3i, 510K approval, December 2021) and included an updated user interface with an online adaptive parallel workflow and updated gating protocols that enable multi-planar tracking capabilities. Specifically, the MR multi-planar tracking capabilities now include anatomical tracking and/or dose-guided tracking for real-time gating. With these novel A3i features, we chose to implement “two planar” tracking for this patient: dose-guided tracking of the GI luminal OAR and anatomical tracking of the target in the sagittal and coronal planes, respectively. Owing to the patient’s history of IBD and its close proximity TO the target volume, duodenum was the prioritized OAR in this case to minimize the risk of acute inflammatory flare-ups or late effects like ulceration, perforation, or fibrosis.

### Online adaptation and isotoxicity planning

Our institutional online adaptive MRgRT workflow has been reported previously (1). Target volumes were rigidly registered, and OARs were deformably registered from the simulation MR to the daily volumetric MR scan. All OARs within 2 cm axially and 3 cm craniocaudally of the PTV were reviewed and manually edited by the radiation oncologist. Manual electron density edits were performed on the deformed CT to match the anatomy of the daily MR scan.

Following segmentation, a predicted plan was calculated using the original plan, generated using the simulated anatomy, and superimposed on the current anatomy and contours of the day to evaluate the indication for adaptation. The predicted plan was considered sufficient if all OARs and target coverage metrics were achieved based on the individualized treatment planning directive. If the predicted plan failed for any metric (i.e., under-coverage of the target volume or overdose to any OAR), an adaptive plan was then generated to meet OAR constraints and/or improve target coverage. The first priority was to ensure that the OAR constraints were met, and target coverage was a secondary goal for isotoxic planning.

### Gated treatment

Real-time tracking was performed on the coronal and sagittal planes. For this particular patient, a novel gating scheme of dose-guided tracking utilized the MRIdian A3i features. To this end, we chose to track the duodenum, the most proximal GI OAR, in the sagittal plane, since it seemed ideal to track the OAR with respect to the pre-specified border of the selected isodose line. Meanwhile, the coronal plane was used to track and ensure the target coverage.

**Figure 2:**
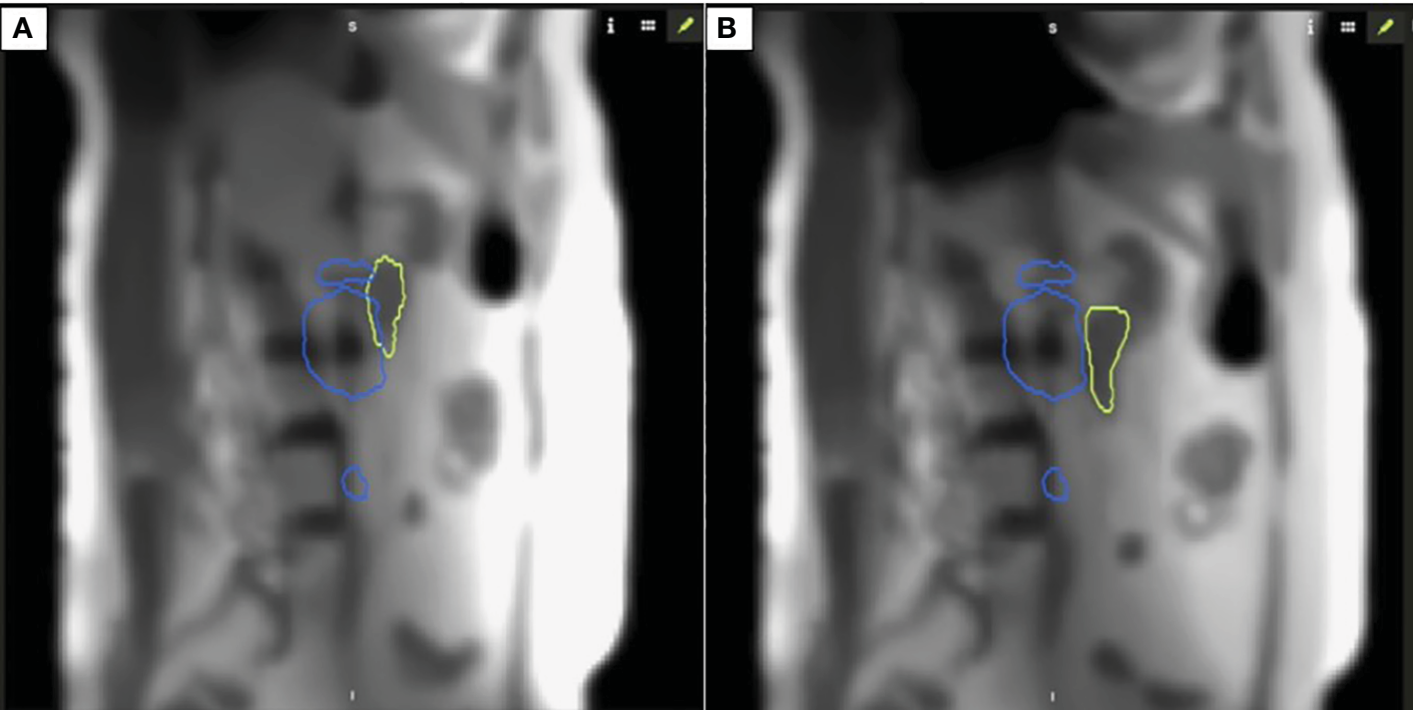
Intrafraction motion with respiration **(A)** demonstrates an overlap between 35 Gy isodose line and duodenum; **(B)** demonstrates separation between the organ-at-risk and respective constraint isodose line when patient is in appropriate breath hold for isotoxic delivery.

Specifically for the sagittal plane tracking, a boundary was defined such that the gating threshold would be that no more than 5% of the duodenum overlapping within the 35-Gy isodose line. This technique was selected for this case since we utilized a dose escalation approach with isotoxicity normalization to the proximal GI luminal OAR, i.e., the duodenum. **Figure 2** demonstrates the intrafraction gating with respiration tracked in the sagittal plane with an earlier overlap between the 35-Gy isodose line and the duodenum (A), and a later separation is observed when the patient is in an adequate breath hold (B).

Additionally, we tracked the gross target on the coronal plane through a tracking structure that approximated the GTV. The target tracking region of interest (ROI) was contoured daily based on the contrast differences of the daily MR to enable the highest accuracy for the deformable image registration algorithm during cine imaging. The sagittal target tracking ROI had a 3-mm boundary margin such that when the deformed tracking ROI moved outside the boundary more than 5%, the beam would automatically be withheld.

**Figure 3:**
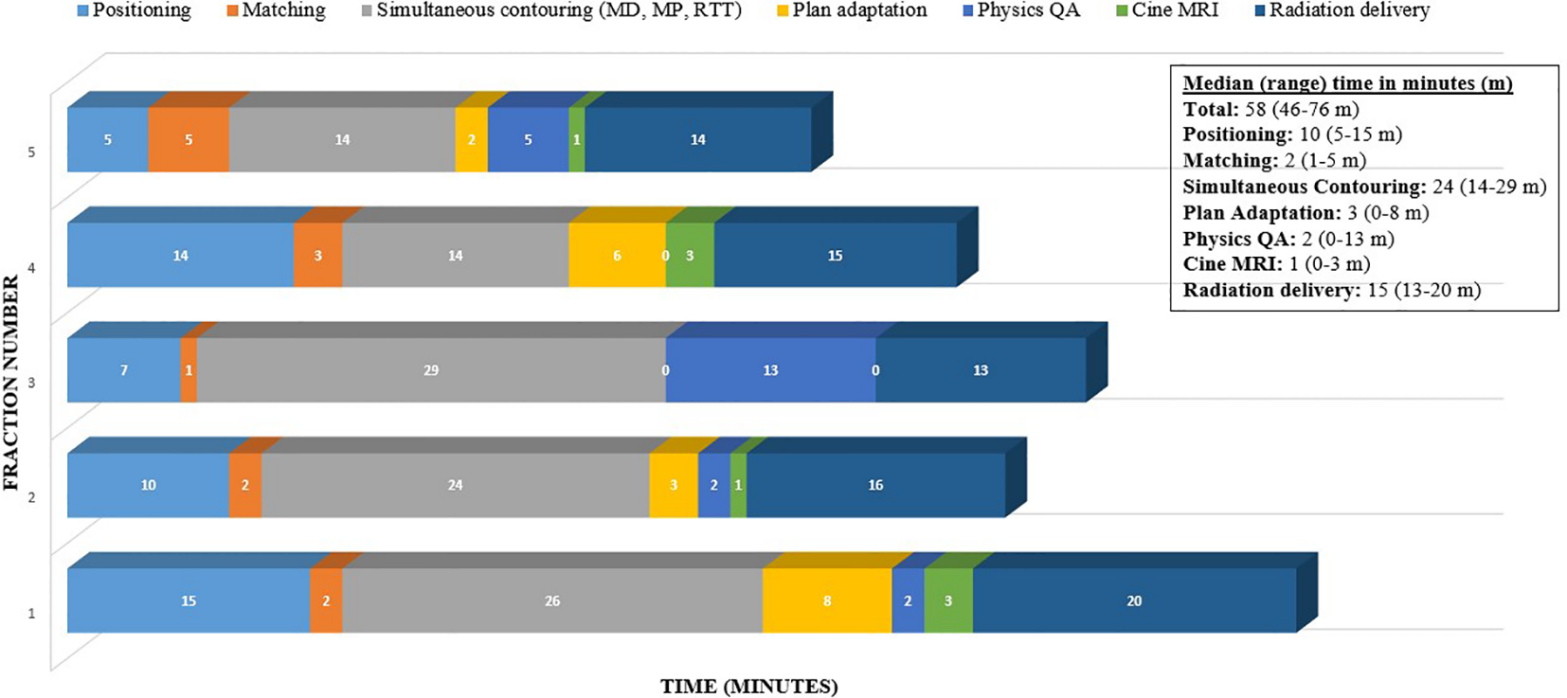
Time (minutes) taken through the workflow for online adaptive MRgRT for this patient case example (5 fractions).

The cine image acquisition was selected as a cartesian sequence at a nominal 4 frames per second (FPS). Note that the cine-temporal resolution is dependent on the FOV. For this case, the achieved frame rate was 3 FPS after FOV adjustments. An FOV of 27.5 × 42 × 0.70 cm and a spatial resolution of 0.35 × 0.35 cm were used, which enabled a frame rate of at least 3 FPS during treatment for each respective plane. Cine imaging was acquired in repeated sequential order between sagittal and coronal orientations. The treatment delivery was a step-and-shoot IMRT, and as such, continuous MR cine imaging occurred for each gantry treatment position. Note that in this isotoxic gating approach, the treatment beam is automatically gated on/off based on the tumor position and duodenum position relative to the high dose gradient. No real-time intra-fraction adaptation was performed based on the duodenum position during treatment delivery. Online adaptation was only performed once per fraction based on the daily volumetric MR scan prior to delivery. **Figure 3** demonstrates the timeline for this patient for each fraction. The median total in-room time was 58 min [range (R): 46–76 min] with the longest on fraction 1 and the shortest on fraction 5. The multi-planar tracking and the breath hold did not substantially increase the treatment time compared to prior literature, with the observed median duration of radiation delivery being 15 min (R: 13–20 min).

## Follow-up

She completed the planned treatment course, with Common Terminology Criteria for Adverse Events (CTCAE) grade 1 nausea being the only acute toxicity. The follow-up PET-CT 3 at months and 6 months post-treatment confirmed complete metabolic and anatomic resolution of the node. With a follow-up at 12 months post-radiation, the patient had durable local control of the treated lesion and the entire PA chain. She has not developed any further late GI toxicity but had one out-of-field failure (left external iliac lymph node). This was also treated with SBRT using MRIdian; however, owing to its quite posterior position and the absence of any abutting GI OAR with respiratory-induced motion, no GI OAR management was needed during cine tracking, and only multi-planar anatomical tumor tracking was used. She continues to have no late toxicity and no evidence of disease on the latest scans in November 2023.

## Discussion

Although multi-object tracking has been technically described, to our knowledge, this is the first report that describes the clinical workflow, successful implementation, and treatment timeline of an isotoxicity approach with dose-guided tracking of a GI OAR (9, 13). With the MRIdian A3i 510K approved for clinical use, several features now facilitate modern SBRT, including multi-planar tracking (up to three planes simultaneously at 4 FPS), dose-guided and anatomical tracking capabilities, and a parallel adaptive workflow that significantly reduces the on-table treatment times. This novel technology was applied to this patient’s case, in which a high-risk OAR was in close proximity to the target in a patient who had a higher predisposition for GI toxicity due to active IBD. The challenge of delivering an ablative dose in this case was that the intrafraction motion of the GI luminal OAR was not synchronous with the target motion. This approach was deemed successful because the patient did not have any significant GI toxicity (only grade 1 nausea), despite being at higher risk and she had complete resolution of the tumor after treatment.

Radiotherapy in the setting of IBD may be at increased risk of severe toxicity, with estimates upwards of 40%–50% in some series (14). In this cohort, 21% of patients required cessation of treatment due to toxicity, which, in the context of curative intent therapy, may result in suboptimal overall survival. There are other cohorts, including a systematic review, suggesting lower rates of toxicity, similar to the baseline risk following pelvic radiation, and some showed a similar risk of toxicity in the setting of IMRT vs. 3D-CRT techniques (15, 16). There is a paucity of data reporting dose constraints for the SBRT technique in the setting of IBD, and for consistency within our institution, similar OAR constraints were used as compared to other indications for adaptive abdominal radiotherapy.

As previously detailed, real-time tracking was performed for the target on the coronal plane and the duodenum, with dose-guided tracking on the sagittal plane. The institutional target coverage and dose constraints for abdominal targets at ablative doses (50 Gy in five fractions) are presented in **Table 1**. A prophylactic dose of 30 Gy was selected in line with the SPARTACUS protocol for elective lymph node coverage (17). With the duodenum adjacent to the target in this case and considering its independent intrafraction motion with respiration, an isotoxicity approach was implemented with the 35-Gy isodose line selected for the dose-guided tracking.

**Table 1:** Institutional target coverage and dose constraints for abdominal targets to ablative dose schedule (50 Gy in five fractions).

Target coverage		
Target	Parameter	Constraint
GTV	D_99%_	≥50 Gy
PTV_50_ (GTV + 3 mm margin)	D_95%_	≥50 Gy
CTV_30_	D_99%_	≥30 Gy
PTV_30_ (CTV + 3 mm margin)	D_95%_	≥30 Gy
OAR constraints		
Organ at risk	Parameter	Constraint
Duodenum	D_0.5cc_	≤35 Gy
	D_0.03cc_	≤38 Gy
Small bowel	D_0.5_	≤35 Gy
	D_0.03_	≤38 Gy
Large bowel	D_0.5_	≤38 Gy
	D_0.03_	≤40 Gy
Stomach	D_0.5_	≤35 Gy
	D_0.03_	≤38 Gy
Spinal canal	D_0.03_	≤20 Gy
Kidneys	D_mean_	≤10 Gy
Liver	D_mean_	≤13 Gy
	V_21 Gy_	≤700 cc
Cauda equina	D_0.03_	≤20 Gy

**Figure 4:**
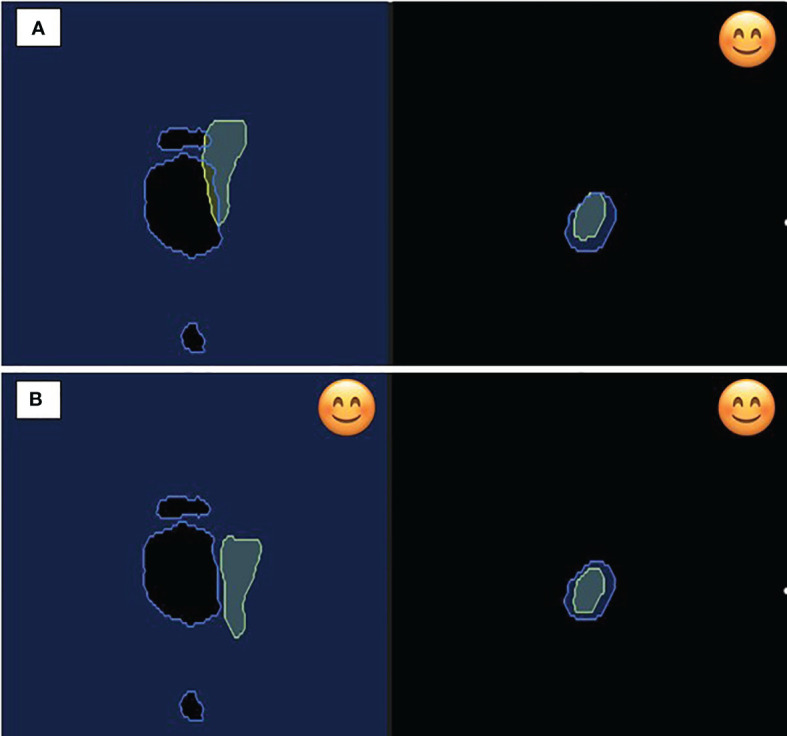
In-room patient monitor demonstrating on the left **(A)** free breathing with overlap between 35 Gy isodose constraint and duodenum; **(B)** breath hold demonstrating appropriate geometry for isotoxic delivery. On the right, figure displays the static target volume demonstrating favorable positioning during both respiration and breath hold. The smile emoji is the feedback to the patient during delivery that the patient is in the correct breath hold position for treatment delivery. Note both the organ-at-risk (left) and the target volume (right) have to be in the correct position for the beam to gate on.

An in-room patient monitor was installed to guide patients regarding the adequacy of their breath hold (**Figure 4**). The monitor displays the real-time motion of the target volume on the right and the OAR on the left. Note that the user has the option to display all planes, a subset of planes, or none. A smiling emoji is displayed on the monitor as real-time feedback to the patient when the breath hold is adequate, and all gating parameters are satisfied.

In our case, we chose gating settings that required both planes to be satisfied for beam-on delivery. As the target and OAR motion are independent of each other with respiration, at times there can be good target coverage but not a separation with the OAR, and *vice versa*. This non-synchronous motion is also demonstrated in **Figure 4**, emphasizing the need for independent, simultaneous tracking of the target and OAR. The treatment beam is automatically gated off at such moments and delivered only when the target is inside and the OAR is outside of the defined boundary.

An increase in the gating events is generally expected, but note that in this case, the target was relatively static, improving the treatment delivery duty cycle. The in-room monitor that provides the visual position of the tumor in real time and feedback to the patient greatly enhanced the adequacy of the breath hold. Moreover, the patients felt actively involved in the treatment process, resulting in improved patient satisfaction, which might have compensated for the treatment time.

AAPM TG 76 recommends the use of appropriate respiratory motion management whenever the motion is >5 mm (18). In clinical practice, image-guided tracking and motion management are restricted to the target, while the OAR is considered secondary; there is no ideal solution to track two ROIs simultaneously in real time. However, SBRT for ablative doses relies on geometric accuracy even when the OAR and target are observed to have independent motion. The technique of multitarget tracking was demonstrated by Liu et al. to simultaneously track targets such as multiple primary lung tumors, prostate, and pelvic lymph nodes (9).

We have adapted this technique to track a target and the adjacent OAR, which have independent respiratory motion to enhance the accuracy of treatment delivery. Conventionally, abdominal and thoracic sites are treated using ITV and PRV, which can lead to inferior target coverage and/or a higher dose to the OAR. While our technique used a GI PRV to define the gradient of the ablative dose of 50 Gy, we performed plan normalization based on the isotoxicity to the nominal GI OAR wall rather than the GI PRV. Without adaptive planning and/or dose-guided tracking, performing isotoxicity to the GI PRV is recommended over the GI OAR wall. Therefore, our approach would rather allow tighter margins for both target and OAR without compromising on target coverage or OAR constraints. The median treatment time of 15 min was equivalent to other MRIdian A3i deliveries of tumor tracking only (19). MRIdian A3i parallel workflow efficiency gains of 30% reduction in online adaptive replanning times have been previously reported (19). The workflow presented here can be adopted for the definitive treatment of any thoracic, abdominal, or pelvic target with dose-limiting OAR in close proximity to ablative doses with an isotoxicity approach.

Beyond this case report, we have implemented dose-guided tracking for all MRgRT patients who have asynchronous motion between the proximal OAR and the tumor. In practice, we have found that the majority of applicable clinical sites are pelvic nodes, in which the target remains static with respiratory motion but the surrounding GI OARs have respiratory-induced motion. Criteria for the section are performed at the time of MRgRT simulation, upon which the geometry of the OAR and target motion is assessed in both the sagittal and coronal cine MR planes. Our future aim is to assess the toxicity profile of patients treated with tumor-based track alone versus dual tumor and isodose-based OAR tracking to quantify the potential reduced toxicity of this method.

## Conclusion

Dose-guided tracking of a GI OAR for isotoxicity delivery is feasible on the MRIdian system for SBRT delivery of a target with an adjacent OAR having non-synchronous respiratory motion to ablative doses. This approach takes advantage of the soft tissue visualization of MR and the real-time multi-planar tracking capabilities of an MR Linac and aids in reducing toxicity while maintaining target coverage.

## Data availability statement

The datasets presented in this article are not readily available because this is a case report. Requests to access the datasets should be directed to the corresponding author.

## Ethics statement

This research was conducted as part of an institutional review board (IRB)-approved retrospective chart review of MR-guided radiotherapy that does not require prospective informed consent. There were no potentially identifiable images or data included in the article.

## Author contributions

SY: Conceptualization, Data curation, Formal analysis, Investigation, Methodology, Writing – original draft, Writing – review & editing. YW: Conceptualization, Investigation, Methodology, Supervision, Writing – review & editing. MC: Investigation, Methodology, Supervision, Validation, Writing – review & editing. NB: Conceptualization, Supervision, Validation, Writing – review & editing. AG: Conceptualization, Supervision, Writing – review & editing. RK: Writing – review & editing. MM: Supervision, Writing – review & editing. KM: Conceptualization, Data curation, Formal analysis, Investigation, Methodology, Software, Supervision, Validation, Writing – original draft, Writing – review & editing.
